# Gaze in the Dark: Gaze Estimation in a Low-Light Environment with Generative Adversarial Networks

**DOI:** 10.3390/s20174935

**Published:** 2020-08-31

**Authors:** Jung-Hwa Kim, Jin-Woo Jeong

**Affiliations:** Department of Computer Engineering, Kumoh National Institute of Technology, Gumi 39177, Korea; junghwa.kim@kumoh.ac.kr

**Keywords:** adversarial network, deep learning, gaze estimation, low-light environment

## Abstract

In smart interactive environments, such as digital museums or digital exhibition halls, it is important to accurately understand the user’s intent to ensure successful and natural interaction with the exhibition. In the context of predicting user intent, gaze estimation technology has been considered one of the most effective indicators among recently developed interaction techniques (e.g., face orientation estimation, body tracking, and gesture recognition). Previous gaze estimation techniques, however, are known to be effective only in a controlled lab environment under normal lighting conditions. In this study, we propose a novel deep learning-based approach to achieve a successful gaze estimation under various low-light conditions, which is anticipated to be more practical for smart interaction scenarios. The proposed approach utilizes a generative adversarial network (GAN) to enhance users’ eye images captured under low-light conditions, thereby restoring missing information for gaze estimation. Afterward, the GAN-recovered images are fed into the convolutional neural network architecture as input data to estimate the direction of the user gaze. Our experimental results on the modified MPIIGaze dataset demonstrate that the proposed approach achieves an average performance improvement of 4.53%–8.9% under low and dark light conditions, which is a promising step toward further research.

## 1. Introduction

Human–computer interaction technologies are becoming increasingly vital for advanced smart interactive systems. To achieve successful and natural interaction between users and devices, the technologies to accurately understand user intent has become all the more important. For example, recent interactive systems attempt to detect user intents expressed in the form of gestures or voice commands using signals from various sensing devices [1,2,3]. More immersive and natural ways to capture user intent include face orientation estimation [4,5,6], body tracking [7,8,9], and estimation of gaze direction [10,11,12,13,14,15,16,17,18,19,20,21]. For example, the authors of [4,5,6] proposed to estimate a driver’s face orientation with color or depth images taken in a specific environment, such as a vehicle, with the aim of preventing traffic accidents. Attempts have been made to detect a human activity by recognizing the joints of a human body with several Kinects for an interactive virtual training environment [8].

Gaze estimation is based on the assumption that the object a user is looking at is the focus of the user’s interest [18]. This technique has been considered one of the most effective indicators with considerable potential to accurately interpret the user intent. Methods for estimating gaze direction can be divided into feature-based methods [10,11,12,13,14] and appearance-based methods [15,16,17,18]. Feature-based methods estimate gaze points using local eye features, such as a the center of the pupil and the cornea, which can be extracted from images of the eye. Therefore, this method generally requires an expensive hardware configuration to obtain high-resolution images of the eye for robust calibration and fine-grained geometry operations. The feature-based methods are divided into three approaches: 3D-model-based, regression-based, and cross-ratio-based approaches. The 3D-model-based approaches compute the gaze points from a 3D geometric eye model [10,11,12,22], regression-based approaches attempt to directly map the gaze points from the local features [13], and cross-ratio-based methods utilize the cross-ratio property of the projective space [14]. Although feature-based methods achieve highly accurate gaze estimation in a controlled set-up, the disadvantages of these methods are their high calibration costs and their requirement for high-resolution images. By contrast, appearance-based methods use an entire eye image itself as input to map the gaze point from the global image feature rather than local eye features. These methods do not require calibration and can be used with a low-cost conventional device such as a webcam; however, their performance is relatively lower than that of feature-based methods [10]. As an example of appearance-based methods, a Gaussian process-based method was proposed for gaze point estimation with images of the entire eye [15]. Recent advances in deep learning techniques, which are able to automatically extract a quality representation from images, have encouraged researchers to attempt an appearance-based approach with various deep neural network architectures. For instance, researchers [16,17,23,24] proposed to apply the convolutional neural network architectures such as VGG-19 [25], RNN [26], and ResNet [27] for gaze estimation using an image of the eye.

**Figure 1:**
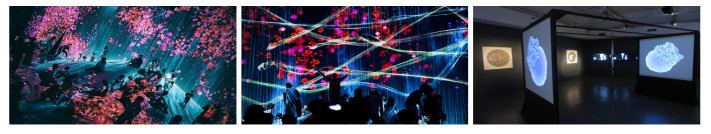
Example of smart interactive environment.

Although various studies concerned with gaze interaction have been reported, the performance of these methods in smart interactive environments under real-life conditions is still not satisfactory. Among the various real-life conditions, this study focuses on the effect of variations in the illumination on the gaze estimation performance. Robust gaze estimation in spaces with low or dark illumination is particularly important for smart interactive environments, such as a digital museums, galleries, or art exhibition halls. For example, the environments shown in Figure 1 usually require interactions with users under low or dark illumination conditions. These visitors would considerably benefit from robust gaze estimation while interacting with smart environments under challenging conditions.

To address this issue, in this paper we discuss a deep learning-based gaze estimation approach robust to challenging lighting conditions. Specifically, we propose an approach to improve the performance of a modern deep learning-based gaze estimation model under low-light conditions. This is achieved by using generative adversarial networks (GANs) to process an image of the eye during the inference phase of the system. The GAN-based method enables us to enhance the appearance of eye images with noise (i.e., low or dark illumination), such that missing information is recovered automatically. The experimental results on the modified MPIIGaze dataset demonstrated that the proposed approach achieved an average performance improvement of 4.53%–8.9%.

The main contributions of this paper are as follows. First, we discuss the way in which low or dark illumination conditions affect the performance of a deep learning-based gaze estimation method. Second, we present the feasibility of gaze estimation under low-light conditions using the GAN-based method, which attempts to adaptively enhance the appearance of low-light eye images, thereby effectively recovering gaze information. Even though GAN-based approaches have been generally used for image generation, reconstruction, and enhancement, how to utilize them for the gaze estimation domain is still not clear. Our analysis will provide various insights on the feasibility of a GAN-based method for gaze estimation in real-life applications. Finally, we conduct various experiments to determine the effects of adopting GAN on the performance of deep learning-based gaze estimation in low-light environments. In particular, we designed various qualitative and quantitative experiments to answer the following research questions.

1. Can a method based on a deep neural network provide a good estimation of gaze points under low-light and dark illumination conditions? This question is to determine the extent to which low and dark lighting conditions affect the performance of a deep learning-based gaze estimation method. To answer this question, we compared the performance of a conventional deep learning-based gaze estimation for normal, low, and dark illuminated images.
2. Could the proposed approach improve the performance of gaze estimation under low illumination conditions? Through this question, we validate the feasibility of the proposed approach which adopts GAN-based image enhancement in the loop. To this end, we conducted experiments to compare the performance of gaze estimation for low-light images between the conventional gaze estimation protocol and the proposed approach.
3. Would a GAN-based adaptive enhancement method be more effective than a simple method involving manual brightness adjustment? The proposed approach is based on a deep adversarial technique to enhance the appearance of low-light eye images. However, a simple image processing method, such as adjusting intensity or gamma values of an image, can be also considered an alternative way to enhance the appearance of low-light images. To show the effectiveness of the proposed approach with a generative adversarial network, we compared the performance of the proposed approach with those of baseline methods.

Finally, we report and discuss the experimental results and present the future research directions.

The remainder of this paper is organized as follows. Section 2 describes related work. In Section 3, we describe the proposed method and Section 4 discusses the experimental results. Finally, we provide our conclusions in Section 5.

## 2. Related Work

In general, image-or vision-based computing and interaction in a low-light environment are problematic in various ways. For example, object detection and recognition under low-light conditions is one of the most important challenges in the field of computer vision. We briefly review previous studies on the enhancement of low-light images and gaze interaction in the real-life environment.

### 2.1. Image Enhancement

Images captured in a low-light environment tend to be characterized by low contrast, low visibility, and contain a large amount of high noise. These characteristics usually have the effect of complicating computer vision tasks such as autonomous driving and object recognition or classification. Attempts to overcome these problems resulted in the development of methods to brighten the image itself on the basis of the Retinex theory [28], which explains the color perception properties of the human vision system. Single-scale Retinex (SSR) achieves an enhanced result using center/surround Retinex, the general form of which is similar to the Difference of Gaussian (DoG), which can be defined by Equation (1),

(1) $$R_{ssr}\left(x,y\right)=log\left(I\left(x,y\right)\right)-log\left(G\left(x,y\right)*I\left(x,y\right)\right),$$

where $R_{ssr}$ is the associated Retinex output, I(x,y) is an image, G(x,y) is a Gaussian function, and ∗ denotes the convolution operation. Multiscale Retinex (MSR) is considered a weighted sum of the outputs of several different SSR outputs [29], which is denoted as follows,

(2) $$R_{msr}\left(x,y\right)=\sum_{i}^{}w_{i}R_{ssr}\left(x,y\right).$$

However, the images enlightened by these algorithms often appear unnatural [29]. To compensate for this problem, the authors of [29] proposed MSR-Net, based on multiscale Retinex, to directly learn the end-to-end mapping between dark-light and normal-light images. This study succeeded in improving the brightness of images by using logarithmic transformation, a convolution operation, and a color restoration process. The authors of [30] proposed Retinex-Net that decomposes the images into reflection and illumination and enhances the brightness of the low-light images. Retinex-Net reconstructs an image with an encoder–decoder network using the adjusted illumination and denoised reflection. A dataset consisting of pairs of low-and normal-light images used for training networks was also constructed. Another attempt to improve the illumination of an image led to the proposal of a global illumination-aware and detail-preserving network (GLADNet) based on an encoder–decoder architecture [31]. This network first calculates the global illumination estimation for an input image. Then GLADNet adjusts the illumination under the guidance of the estimation and reconstructs the details by concatenating the intermediate result with the original input.

In addition to the studies based on the Retinex theory, convolutional neural network (CNN)-based studies have also been presented. A novel image descriptor using U-Net and a VGG network was proposed to improve the image retrieval performance under various lighting conditions [32]. To this end, they constructed a dataset consisting of images of day–night pairs of images. In addition, a fully convolutional network based on end-to-end training was proposed to enhance the brightness of low-light images [33]. The authors of [33] also constructed a dataset comprising raw images taken in a low-light environment to compensate for the lack of large-scale low-light images suitable for training or testing purposes.

The aforementioned studies commonly required datasets containing pairs of low-light and normal images. However, it is difficult to capture a large number of these image pairs. Therefore, various studies have attempted to enhance the brightness of low-light images using a GAN-based approach [34,35,36]. For example, a method named EnlightenGAN was proposed to enhance the brightness of low-light images when a set of low-light and normal image pairs is unavailable [36]. EnlightenGAN has the following procedure. First, the low-light image is inputted to a generator consisting of attention-guided U-Net [37]. Subsequently, a global and local discriminator architecture is used to distinguish between real images and images artificially generated from low-light images. Their experimental results showed EnlightenGAN framework to be effective in the domains of face, building, and natural scene.

### 2.2. Gaze Estimation in a Real-Life Environment

As the need for smart interaction under real-life conditions increases, gaze estimation under real-life conditions (e.g., long-distance, mobile, and low-light) has become more important as well.

#### 2.2.1. Long-Distance Set-Up

As shown in Figure 1, advanced smart interactive environments typically require a set-up of long-distance between users and displays, which makes it comparatively more difficult to accurately estimate the gaze direction of a user. Efforts to solve this problem resulted in the proposal of a public dataset consisting of eye images that were captured by a camera 1.0−1.5m away from the user in both indoor and outdoor environments [38,39]. Subsequently, a method was proposed for estimating the gaze of a user who posed naturally in a laboratory with normal lighting conditions [38]. A set of motion capture systems, eye tracking glasses, and RGB-D cameras was used to discover the mapping between the gaze points and the user’s face image. Finally, they estimated the gaze direction using an image of the face and images of the left and right eye. In [39], a dataset was constructed by capturing users at various angles and in various settings in an outdoor environment using an AprilTag [40] and MegaPixel cameras. The AprilTag was used to specify the target the user was required to look at and MegaPixel cameras were utilized to capture 360-degree images of the user. The constructed data were fed into a long short-term memory (LSTM) network to obtain the final gaze direction as output.

#### 2.2.2. Mobile Environment

Conventional gaze interaction studies usually collected gaze data by a set of cameras installed at a fixed location, such as at the bottom of a monitor [10,16,41], or standing on the floor or mounted on a wall [38,42]. Recently, attempts were made to collect gaze data by using portable devices, such as mobile phones and tablets. For example, gaze data were collected in a daily-life environment using a mobile tablet [43,44]. For data collection, participants of [43,44] were instructed to gaze at the point displayed on a tablet screen. The researchers of [43] used a CNN to estimate the gaze direction using the left eye, right eye, facial images, and face grid information. Others [44] cropped the images of the left and right eyes to extract a set of eye features, such as the histograms of oriented gradients (HOG) [45]. The extracted features were used to predict the final gaze point by using various regression algorithms, such as k-nearest neighbor (k-NN), and random forest (RF).

#### 2.2.3. Low-Light Conditions

As mentioned earlier, current smart interactive environment usually interacts with users under low or dark illumination conditions. However, it is difficult to estimate the user’s gaze direction or orientation under such a challenging condition. Several studies endeavoring to address this limitation have been reported. In [17], a laptop webcam was used to construct a dataset in both indoor and outdoor environments. It was proposed that gray scaling and histogram equalization be applied to images and a classification model be trained using a set of synthesized eye images by varying the illumination. The authors generated cropped images of eyes using eye location extracted by facial landmarks and produced head pose vectors from 3D head rotation values through a data normalization process. Afterward, the cropped eye images and head pose vectors were inputted into a CNN model to estimate the final gaze direction. A method to enhance the low performance of gaze estimation under low illumination conditions with an infrared camera was proposed [41]. The gaze direction was estimated using the infrared light points reflected in the pupil. In this way, the gaze features extracted from each camera were fused to estimate the user’s final gaze direction. On the other hand, the authors of [17,38] tried to apply gray-scale conversion and histogram equalization to address the gaze estimation problem under various lighting conditions.

In summary, a number of studies have been conducted to overcome these challenging conditions (i.e., a long-distance set-up, mobile environment, and low-light conditions). In this study, we focus on addressing the problem of gaze estimation under low-light conditions. Although previous studies were also concerned with this problem, several aspects thereof have not yet been satisfactorily resolved and could be seen as limitations: (i) an additional hardware set-up (e.g., infrared LEDs) is required, (ii) extra training data such as synthesized images are required, and (iii) the number of test images captured in both low and dark illumination continues to remain limited. To overcome these limitations, we decided to employ a GAN-based approach, which is well known to reconstruct an image in the facial, building, and natural scene domain, to an image in the eye and gaze estimation domain. In particular, we investigated the feasibility of using the GAN-based approach for gaze estimation under low-light conditions.

## 3. Proposed Method

**Figure 2:**
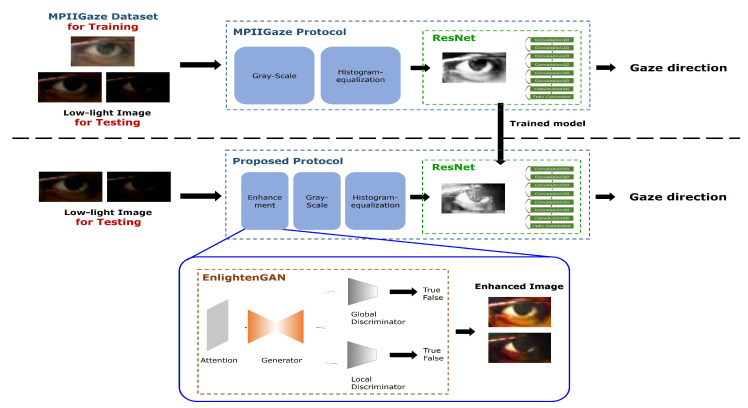
Overall workflow of the proposed approach.

The overall gaze estimation process of this study follows the MPIIGaze protocol [17], as shown in the top of Figure 2. The MPIIGaze protocol consists of the following steps: first, eye images of the MPIIGaze dataset captured under various lighting conditions are preprocessed by gray-scale conversion and histogram equalization. As a result of preprocessing, a set of binary images of the eye is obtained from the raw eye images. Afterward, MPIIGaze is used to train a deep neural network using the binary images and estimate the user’s gaze direction.

The proposed protocol extends the original MPIIGaze protocol by adding an enhancement process to recover the appearance of low-light eye images during the test phase. The overall workflow of the proposed protocol is shown in the lower panel of Figure 2. Unlike the original MPIIGaze protocol, the proposed protocol contains an additional enhancement module before the gray scaling and histogram equalization module. The enhancement module recovers the appearance of low-and dark-light eye images using a GAN-based framework. During the test phase, given a low-light eye image, the appearance of the image is recovered by using the GAN-based framework, following which gaze estimation is conducted. To simulate eye images with low-and dark-light conditions, we constructed an additional dataset and used this dataset to observe the changes in the performance of the original MPIIGaze protocol. Additional details on the enhancement module are presented in Section 3.2. It should be noted that additional set-ups (e.g., hardware calibration, more training samples, and training parameter optimization) are not required for the training phase. Finally, we verified the extent to which the proposed approach affects the performance of the original MPIIGaze protocol on gaze estimation under low-and dark-light conditions by carrying out various experiments.

### 3.1. Dataset

**Figure 3:**
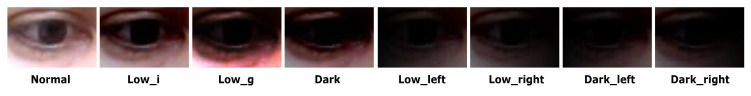
Examples of low and dark illuminated test sets.

In this study, we used the MPIIGaze dataset [17] to evaluate the gaze estimation performance under various lighting conditions. This dataset contains a total of 213,659 images collected from 15 participants using conventional laptop webcams. For our experiment, the illuminance of the images in the MPIIGaze dataset was programmatically adjusted to simulate low and dark illumination conditions. According to the MPIIGaza protocol, 1500 images of left eyes and 1500 images of right eyes per subject, for a total of 3000 images are randomly sampled. In total, 45,000 images were collected from 15 people. Based on this dataset, we constructed various test sets to evaluate the performance of the original MPIIGaze pipeline and the proposed approach. The constructed test set includes eye images with various lighting conditions as shown in Figure 3: (1) The original MPIIGaze test set (Normal), (2) low and dark illuminated test sets ($Low_{i}$, $Low_{g}$, and Dark), and (3) low and dark illuminated images with directional lights ($Low_{left}$, $Low_{right}$, $Dark_{left}$, and $Dark_{right}$).

**Figure 4:**
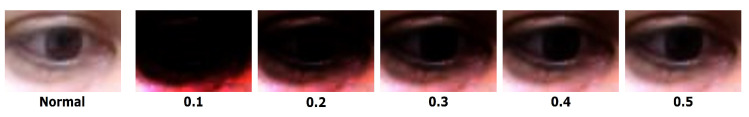
Examples of gamma-corrected images.

The $Low_{i}$ and Dark test sets were generated by uniformly decreasing the intensity of the Normal image (i.e., decreasing the intensity value of each pixel in the images by 70 and 110, respectively). The $Low_{g}$ test set is an additional type of a low-illuminated image set generated by applying gamma correction to Normal images. Here, a gamma-corrected output image *O* can be obtained by $O=\;I^{\left(1/G\right)}$ × 255, where *I* is an input image and *G* is the gamma value. When the gamma value is set to be greater than 1, the brightness of an image increases accordingly. Similarly, when the value of gamma is less than 1, the image becomes darker. Figure 4 shows the extent to which gamma correction at different levels (e.g., 0.1–0.5) affects the appearance of the original image. The closer the gamma value is to zero, the more the eye becomes unrecognizable. As a result of gamma correction, we found that the appearance of images with a gamma value of 0.1 and 0.2 is exceptionally dark and that of images with a gamma value of 0.4 and 0.5 does not differ significantly from the Normal image. Therefore, we constructed the $Low_{g}$ test set by correcting the images with a gamma value of 0.3.

**Figure 5:**
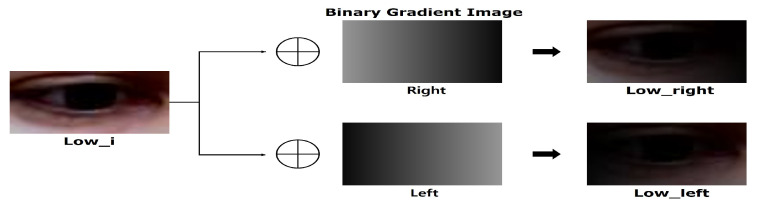
Generating process of directional light images.

**Figure 6:**
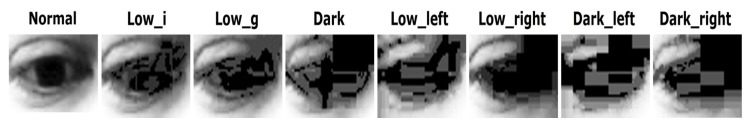
Binary images of low and dark illuminated test sets.

Finally, we constructed additional test sets to simulate the positional change of the lighting source. The images in the $Low_{left}$ and $Dark_{left}$ ($Low_{right}$ and $Dark_{right}$) were simulated as though the lighting source is positioned to the left (right) of the subject. The $Low_{left}$ and $Low_{right}$ test sets were generated by merging the $Low_{i}$ test sets and binary gradient images that become darker either to the left or the right, respectively. Similarly, the $Dark_{left}$ and $Dark_{right}$ test sets were generated by merging dark illuminated images and binary gradient images. The merging process is shown in Figure 5. Similar to the original MPIIGaze protocol, an image preprocessing pipeline consisting of histogram equalization and gray scaling was also applied to our additional test sets before training and testing the network. Examples of the binary images of the eye obtained after proceeding through the preprocessing pipeline are presented in Figure 6. The binary images of the $Low_{i}$ and $Low_{g}$ test sets contain a certain amount of noise, but the eye is still recognizable. However, the eyeball is hardly recognizable in the binary images in the Dark test set. Finally, the binary images obtained from the low and dark illuminated images with directional lights (i.e., $Low_{left}$, $Low_{right}$, $Dark_{left}$, and $Dark_{right}$) commonly contain a large number of dark holes, which present yet another challenge for estimating the gaze directions.

**Figure 7:**
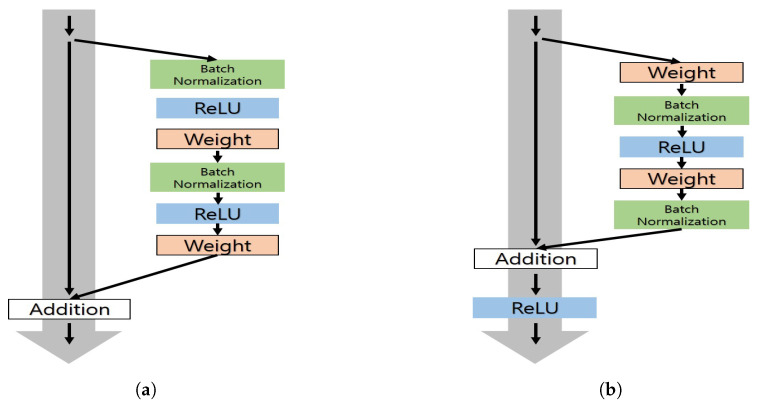
Comparison of (**a**) ResNet pre-act architecture and (**b**) original ResNet architecture.

To implement deep learning-based gaze estimation, we used the preactivation version of the ResNet architecture, which consists of seven convolutional layers and a single fully connected layer [46]. The preactivation version of ResNet (see Figure 7a) is a variation of the original ResNet (see Figure 7b) and is known to improve the performance of the original ResNet by placing an activation layer before the weight layer.

As mentioned earlier, the input for the network used in this study is a binary image of the eye, sized of 60 × 36, and processed by the MPIIGaze preprocessing pipeline (i.e., gray scaling and histogram equalization). This input image was used together with a 3D head pose vector when training or testing the network. Finally, the output produced by ResNet is a 3D gaze angle vector that includes the yaw, pitch, and roll values of the gaze angle. We used the cosine angular distance as a loss function that measures the difference between the ground-truth gaze angle vector and the predicted gaze angle vector. This loss is referred to as the gaze angular error in the experimental section.

### 3.2. Enhancing Eye Images with GAN

**Figure 8:**
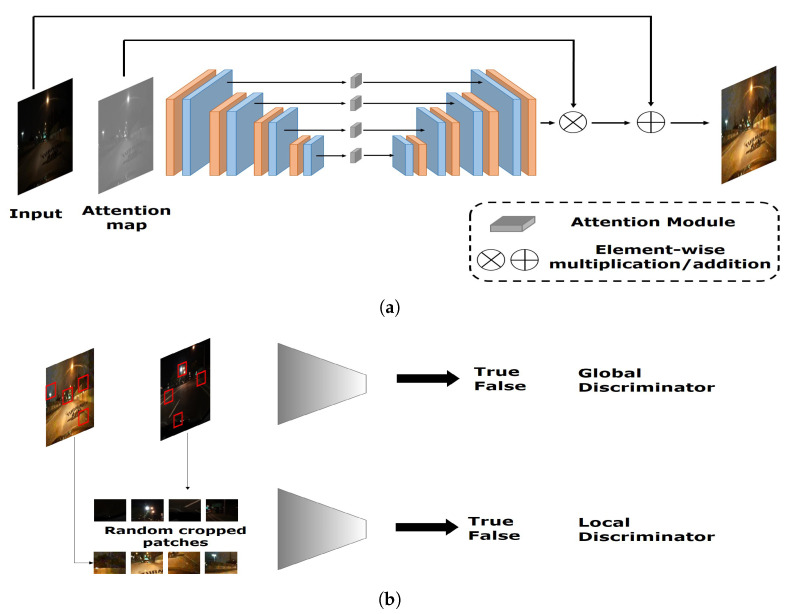
Architecture of EnlightenGAN framework: (**a**) generator and (**b**) discriminators.

To obtain usable images of the eye with low and dark illuminations without any additional hardware set-up and training images, we propose using a GAN to recover near-original images of the eye from the low and dark illuminated eye images. Our assumption is that the images recovered by the generative approach would retain more informative data than images captured in low or dark illumination, and that this would improve the gaze estimation performance. To this end, we exploited the EnlightenGAN framework with the goal of improving the brightness of a low-light image by training with normal-light images only (i.e., without requiring pairs of low-light and normal images). The architecture of the EnlightenGAN framework is shown in Figure 8. Similar to standard GAN-based architectures, EnlightenGAN has two components: a generator and a discriminator.

The generator of EnlightenGAN serves to perform image generation and enhancement with the U-Net architecture [37] (see Figure 8a). The generator accepts the low-light image and its corresponding attention map as its input. The attention map is simply generated based on the intensity of the illumination of the input image. According to the U-Net architecture, EnlightenGAN first generates a set of feature maps in multiple scales. The attention map is resized to fit each feature map and multiplied with all the intermediate feature maps. Subsequently, element-wise multiplication and addition operations are conducted to generate the enhanced image. This image is then sent as input to the discriminator of EnlightenGAN, which consists of both global and local discriminators to process spatially varying light conditions. The global discriminator discriminates the entire image, whereas the local discriminator discriminates a set of local and partial information of an image using random patches cropped from the image. An illustrative example of the discriminator is shown in Figure 8b.

Finally, the loss functions for the generator *G* and the global discriminator *D* are defined as follows [36],

(3) $$L_{D}^{Global}=E_{x_{r}∼P_{real}}\left[{\left(D_{Ra}\left(x_{r},x_{f}\right)-1\right)}^{2}\right]+E_{x_{f}∼P_{fake}}\left[D_{Ra}{\left(x_{f},x_{r}\right)}^{2}\right],$$

(4) $$L_{G}^{Global}=E_{x_{f}∼P_{fake}}\left[{\left(D_{Ra}\left(x_{f},x_{r}\right)-1\right)}^{2}\right]+E_{x_{r}∼P_{real}}\left[D_{Ra}{\left(x_{r},x_{f}\right)}^{2}\right],$$

where $D_{Ra}$ is the function of the discriminator, and $x_{r}$ and $x_{f}$ are sampled from the real and fake distributions. The loss functions for the local discriminator and generator are defined as follows,

(5) $$L_{D}^{Local}=E_{x_{r}∼P_{real-patches}}\left[{\left(D\left(x_{r}\right)-1\right)}^{2}\right]+E_{x_{f}∼P_{fake-patches}}\left[D{\left(x_{f}-0\right)}^{2}\right],$$

(6) $$L_{G}^{Local}=E_{x_{r}∼P_{fake-patches}}\left[{\left(D\left(x_{f}\right)-1\right)}^{2}\right].$$

A new loss function $L_{SFP}$ was also introduced to preserve the self-feature of an image after the enhancement [36], which is defined as follows,

(7) $$L_{SFP}\left(I^{L}\right)=\frac{1}{W_{i,j}H_{i,j}}\sum_{x=1}^{W_{i,j}}\sum_{y=1}^{H_{i,j}}{\left(\phi_{i,j}\left(I^{L}\right)-\phi_{i,j}\left(G\left(I^{L}\right)\right)\right)}^{2},$$

where $I^{L}$ is an input low-light image, $G(I_{L})$ is the enhanced output of the generator, $\phi_{i,j}$ is a feature map extracted from the pre-trained VGG-16 model [25], *i* denotes the *i*th max pooling, *j* denotes the *j*th convolutional layer after the *i*th max pooling layer, and *W* and *H* are the width and height of the extracted feature maps, respectively. The final loss of EnlightenGAN is then computed by

(8) $$Loss=L_{SFP}^{Global}+L_{SFP}^{Local}+L_{G}^{Global}+L_{G}^{Local}.$$

**Figure 9:**
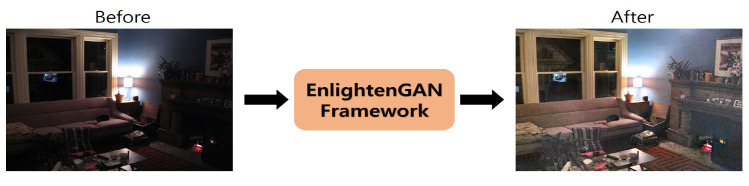
Example of images in the furniture domain reconstructed by EnlightenGAN.

An example of an image from the furniture domain enhanced by the EnlightenGAN framework is shown in Figure 9. As shown in the figure, the low-light image becomes more visually recognizable after it was brightened by the EnlightenGAN framework. Previous experimental results [36] showed that the EnglighenGAN framework was applicable to various domains, such as nature, buildings, and faces. We expected the EnlightenGAN-based approach to be effective in the eye domain because it was well generalized in various domains, even though the enhancement of images of eyes has never been tried or validated yet. Therefore, we conducted various experiments to determine whether the proposed approach with EnlightenGAN would be effective in the eye domain for images captured under low-light conditions. The experimental results are reported in detail in Section 4.

## 4. Experiments and Discussion

The experiments were designed to answer the following research questions.

(1) Can a method based on a deep neural network provide a good estimation of gaze points under low-light and dark illumination conditions? We need to determine the extent to which low and dark lighting conditions that are commonly found in current interactive environments affect the overall performance of a deep learning-based gaze estimator. To answer this question, we compared the performance of the MPIIGaze protocol in terms of its estimation accuracy for normal, low, and dark illuminated images. As discussed in Section 3.1, the images captured under low/dark-light conditions tend to have more noise than those captured under normal-light conditions. According to this analysis, we expect that the performance of a conventional deep learning-based gaze estimation will be degraded (H1).

(2) Could the proposed approach improve the performance of gaze estimation under low illumination conditions? We then need to validate the feasibility of the proposed approach, which adopts GAN-based image enhancement in the loop to process low-light conditions. As described in Section 3.2, the proposed approach attempts to enhance the low-light eye images with a generative approach to recover information loss. Therefore, we expect that the proposed approach will perform better than the original MPIIGaze protocol (H2). Furthermore, we observed that much more information loss (e.g., noise, dark holes, etc.) happens to dark-illuminated images as illustrated in Figure 3 and Figure 6. Therefore, we hypothesize that the dark images will benefit more from the proposed approach than low-light images (H3).

(3) Would a GAN-based adaptive enhancement method be more effective than a simple method involving manual brightness adjustment? Indeed, the brightness of an image can be easily adjusted by manually correcting the intensity or gamma values as described earlier. Therefore, one would imagine that increasing the intensity value would be sufficient to brighten the image and that adopting a deep learning-based solution for this purpose would be unnecessarily excessive. To address this doubt, we demonstrate the difference between the proposed method and various baseline methods and compare the performance of the proposed approach with those of these baselines. As introduced in Section 3.1, the test sets contain eye images with various illumination conditions; however, manual intensity or gamma correction methods cannot work in an adaptive way, which is expected to decrease the performance. Based on this, we expect that the baseline methods will generally fail to effectively estimate the gaze direction (H4). However, we also hypothesize that some of the baseline methods can work better than the original MPIIGaze protocol which does not apply any image enhancement method (H5).

The experiments were conducted on a high-end server equipped with two NVIDIA RTX 2080 Ti GPUs, 128 GB RAM, and an Intel i9-7920X CPU. We used the PyTorch [47] framework for the implementation of the MPIIGaze and EnlightenGAN frameworks. The ResNet network was trained for 40 epochs with a batch size of 32 and an SGD optimizer. Image processing, such as intensity and gamma correction, was carried out using the Python PIL library [48]. In our experiment, all the performance evaluations were conducted based on leave-one-subject-out validation with ten repetitive experiments.

### 4.1. Deep Learning-Based Gaze Estimation under Low and Dark Illumination Conditions

In this section, we answer our first research question: Can a method based on a deep neural network provide a good estimation of gaze points under low-light and dark illumination conditions?

First, we computed the gaze angular error of the MPIIGaze protocol on the Normal test set. Afterward, we evaluated the performance of the MPIIGaze protocol using the various aforementioned low and dark-illuminated test sets (i.e., $Low_{i}$, $Low_{g}$, Dark, $Low_{left}$, $Low_{right}$, $Dark_{left}$, and $Dark_{right}$). In this experiment, an enhancement method was not applied when testing the network. The results of this experiment would enable us to determine the effect of low and dark illumination conditions on the performance of the gaze estimation method based on deep learning.

The performance of the MPIIGaze protocol on the low and dark illuminated test sets is reported in Table 1. The reported values are the averaged angular error measured from all the participants in each test set. The results in the table show that low and dark illumination conditions drastically degrade the performance of the MPIIGaze protocol. Compared to the Normal test set (i.e., 5.76), the average gaze angular error on the low illuminated sets increased to 9.71–10.4 degrees (i.e., 9.71 for $Low_{g}$, 9.9 for $Low_{i}$, 10.4 for $Low_{left}$, and 10.2 for $Low_{right}$). In addition, the mean angular error on the dark illuminated test sets increased to approximately 10 degrees (i.e., 10.56 for Dark, 10.4 for $Dark_{left}$, and 10.22 for $Dark_{right}$). This implies that low and dark illumination conditions cause the performance of ResNet to decrease by as much as 80.5% (i.e., from 5.76 to 10.4 on $Low_{left}$) and 83.3% (i.e., from 5.76 to 10.56 on Dark), respectively. From these results, we showed that H1 can be confirmed. Furthermore, the findings for the test sets containing images with directional light reveal that the brightness level does not affect the performance. For example, both $Low_{left}$ and $Dark_{left}$ caused angular errors of 10.4. Similarly, both $Low_{right}$ and $Dark_{right}$ caused angular errors of 10.2. For these test sets, the direction of the light mainly affected the gaze estimation performance.

By contrast, the performance on $Low_{i}$, $Low_{g}$, and Dark is largely dependent on the brightness level. As indicated in Table 1, when tested with $Low_{i}$, $Low_{g}$, and Dark, the average angular error increased by 4.14, 3.95, and 4.8 degrees compared to Normal, respectively. The MPIIGaze protocol experienced the highest performance reduction on Dark. This result is consistent with our observation that, in the binary images of the eye (Figure 6), the appearance of the eyeball in Dark is much more noisy and thus, less recognizable than in $Low_{i}$ and $Low_{g}$. Finally, considering the performance difference between $Low_{i}$ and $Low_{g}$ (i.e., 9.9 on $Low_{i}$ and 9.71 on $Low_{g}$), it can be inferred that gamma correction retains more of the available gaze information.

### 4.2. Performance Improvement of the Proposed Approach

The next experiment was carried out to answer our second research question: *Could the proposed approach improve the performance of gaze estimation under low-illumination conditions?* For this experiment, we evaluated the performance of the proposed approach that applied GAN-based image enhancement before the image preprocessing step and gaze estimation using ResNet (see Figure 2).

**Figure 10:**
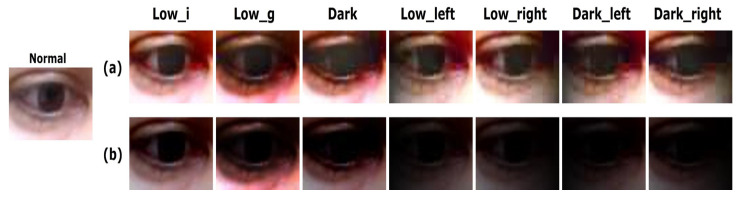
Comparison of images of the eye: (**a**) Images enhanced by EnlightenGAN. (**b**) Original low and dark illuminated images.

**Figure 11:**
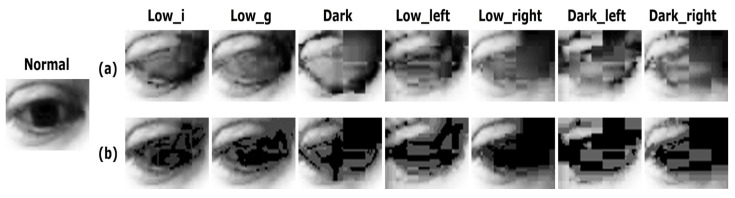
Comparison of binary images of the eye: (**a**) Images enhanced by EnlightenGAN. (**b**) Original low and dark illuminated images.

First, we describe the enhancement of low and dark illuminated images of the eye with the GAN-based enhancement method. Illustrative examples of low-light eye images and their enhanced versions are shown in Figure 10. As shown in Figure 10a, the images that were enhanced by the generative approach definitely have brighter illumination with more well-defined eye and pupil boundaries when compared with the original low and dark illuminated images shown in Figure 10b. Figure 11 represents the difference between the binary images of the enhanced eye images (Figure 11a) and the original low/dark illuminated images (Figure 11b). These results show that the binary images of the original low/dark illuminated images are generally noisier compared to the binary images of the enhanced eye images. Specifically, the binary versions of the original low and dark illuminated images contain more dark holes, which may lead to the loss of additional information that would be useful for gaze estimation. In particular, the binary image of the original image in Dark appears to be partially cropped, with the result that the eye boundary is less well-defined compared to the binary version of the enhanced image of the eye. A similar phenomenon is observed in the case of the binary images of test sets with directional lights. Based on this qualitative analysis, gaze estimation using images enhanced by the generative approach would be expected to yield superior performance.

Next, we present our quantitative analysis of the performance of the proposed approach. The performance of the MPIIGaze protocol and that of the proposed approach on low and dark illuminated test sets is compared in Table 2. First, as summarized in the third and fourth row of the table, the performance of the proposed approach on $Low_{i}$, $Low_{g}$, and Dark sets improved by up to 4.53%–8.9% compared to that of the MPIIGaze protocol. Specifically, the proposed method achieved a performance gain of 0.44–0.48 degrees (4.53%–4.8%) on the low illuminated test sets (i.e., from 9.9 to 9.42 on $Low_{i}$ and from 9.71 to 9.27 on $Low_{g}$) and 0.95 degree (8.9%) on the dark illuminated test set (i.e., from 10.56 to 9.61 on Dark), which confirms H2. This also indicates that the performance improvement with the proposed method increases as the illumination of images decreases; therefore, our hypothesis H3 can be confirmed. This is consistent with our analysis of the binary images of the eye obtained from the original images and those obtained from the images enhanced by EnlightenGAN. As mentioned earlier, we found that the binary version of the original image in Dark appears to be partially cropped, thereby rendering the appearance of the eye ambiguous and incomplete. We believe that this adversely affected the performance of the MPIIGaze protocol.

Second, the proposed method was also able to effectively process the directional lighting test sets. The proposed method achieved a performance improvement of 6.8%–7.15% for the low-light directional sets (i.e., from 10.4 to 9.69 on the $Low_{left}$ test set and from 10.2 to 9.47 on the $Low_{right}$ test set). In addition, a performance improvement of 6.75%–7.01% was obtained for the dark-light directional sets (i.e., from 10.22 to 9.53 on the $Dark_{right}$ test set and from 10.4 to 9.67 on the $Dark_{left}$ test set). These results validated our hypothesis H2; however, hypothesis H3 was not met. There was no significant difference in performance improvement between low-light directional sets and dark-light directional sets. Similar to the experimental result reported in Section 4.1, however, the performance differs slightly depending on the direction of light. Similar to the MPIIGaze protocol, the proposed method is more effective on the right directional test sets than the left directional test sets. As shown by the results in Table 2, the mean angular error of the proposed method on the left directional test sets is 9.67–9.69 degrees (i.e., $Low_{left}$ and $Dark_{left}$), which is larger than on the right directional test sets of 9.47–9.53 degrees (i.e., $Low_{right}$ and $Dark_{right}$). This result implies that the binary image of the eye of the right directional sets preserve more informative data than the left directional sets even though the appearance of the eyeball is still not clear. Conversely, we also found that the brightness level does not affect the performance of the proposed method on the directional light test sets. For example, the proposed method has an angular error of 9.67–9.69 on $Dark_{left}$ and $Low_{left}$ vs. 9.47–9.53 on $Low_{right}$ and $Dark_{right}$. This result is also consistent with our previous observation that the performance of the MPIIGaze protocol is not dependent on the brightness level when processing the directional light test sets.

In summary, we found that the proposed method outperformed the MPIIGaze protocol under various challenging conditions. The performance improvement can be attributed to the capability of the proposed approach to recover missing information from low and dark images of the eye as a result of enhancement in a generative way. More importantly, the performance improvement could be achieved without any additional hardware set-up or training.

### 4.3. Comparison with Baseline Methods

In this section, we provide the answer to our final research question: Would a GAN-based adaptive enhancement method be more effective than a simple method involving manual brightness adjustment?

Our experimental results showed that the gaze estimation performance can be improved under challenging conditions when using the proposed method with EnlightenGAN, which can recover the features of low and dark illuminated eye images. However, it would also be reasonable to consider simple image processing methods, such as intensity adjustment or gamma correction, to enhance the low-light image. To address this consideration, we conducted experiments to compare the gaze estimation performance between the proposed method and the baseline methods, with the latter methods consisting of both intensity adjustment and gamma correction methods. The list of baseline methods is as follows.

1. Gamma correction methods: $G_{low}$, $G_{mid}$, and $G_{high}$
  - Set the gamma value to be 1.5, 2, and 3
2. Intensity adjustment methods: $I_{low}$, $I_{mid}$, and $I_{high}$
  - Increase the intensity by 40, 70, and 110

**Figure 12:**
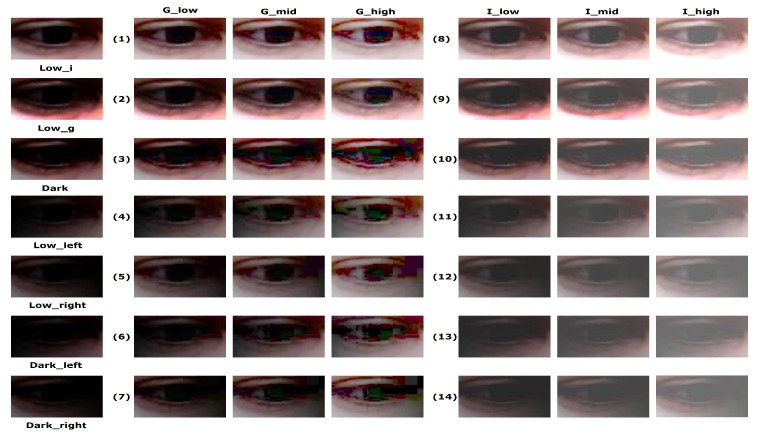
Examples of images modified by the baseline methods.

Examples of images modified by the baseline methods can be seen in Figure 12. The illumination of modified images of the eye has been changed; however, the quality of the result differs significantly depending on the particular method that was used. For example, the images that were modified by adjusting their intensity (i.e., $I_{low}$, $I_{mid}$, and $I_{high}$) are more blurry than those processed with gamma correction (i.e., $G_{low}$, $G_{mid}$, and $G_{high}$). In particular, the images that were modified by adjusting their intensity appear to be merely hazy, rather than brighten. Conversely, the gamma correction methods successfully enhanced the images in $Low_{i}$ and $Low_{g}$, such that the resultant images of the eye contained little noise and had well-defined appearances (see row (1) and (2) in Figure 12). However, these methods failed to enhance the images in the remaining test sets (see row (3)–(7) in Figure 12). In addition, for all the gamma correction methods, the larger the gamma value the noisier the appearance became. Based on this qualitative result, certain baseline methods would be expected to have a chance to outperform the MPIIGaze protocol.

Next, we report the quantitative results of the comparative experiments. The performance of the proposed method is compared with that of the baseline methods in Table 3. We first discuss the results of the gamma correction methods and then analyze the performance of the intensity-based methods.

First, the average angular error of the baseline methods that employ gamma correction decreased by 0.07–0.13 degrees compared with the results obtained by using MPIIGaze. In particular, $G_{mid}$ achieved the highest performance gain among the gamma correction methods. However, it should be noted that the average angular error of the different gamma correction methods does not vary much (i.e., the average error ranges from 10.06 to 10.12). In addition, as observed from our qualitative analysis, the gamma correction methods performed well on the $Low_{i}$ and $Low_{g}$ test sets (i.e., the angular error was in the range 9.56 to 9.88); however, these methods were ineffective on the Dark test set (i.e., the angular was in the range of 10.39 to 10.48). The gamma correction methods were similarly ineffective on the directional light test sets (i.e., the angular error was 10.1–10.36). Nevertheless, the methods based on gamma correction outperformed the MPIIGaze protocol. Specifically, the average angular error decreased by 0.02–0.15 degrees, 0.08–0.17 degrees, and 0.06–0.25 degrees for $Low_{i}$ and $Low_{g}$, Dark, and the directional light test sets, respectively.

Even though the gamma correction methods slightly outperformed MPIIGaze, they still performed worse than the proposed method. The proposed method achieved an average performance improvement of 5.67%–6.3% over gamma correction methods for all the cases. For example, gamma correction produced errors in the range 9.56 to 9.88 degrees on the $Low_{i}$ and $Low_{g}$ test sets, whereas the errors of the proposed method were between 9.27 and 9.42. Furthermore, the performance of the gamma correction method became much worse on the Dark and directional light sets (i.e., 10.25 degrees on average), whereas the errors of the proposed increased slightly to 9.59 degrees on average. This means that the proposed method can successfully recover information lost under low-light conditions compared with the gamma correction methods.

Second, we discuss the performance of the methods based on intensity adjustment (i.e., $I_{low}$, $I_{mid}$, and $I_{high}$). The results in Table 3 indicate that the methods based on intensity adjustment delivered the lowest performance (i.e., the angular error was in the range 10.11 to 10.75) among the enhancement methods. In particular, the proposed method outperformed the intensity adjustment methods with decreased angular errors of 0.59–1.23 degrees (i.e., performance improvement of 5.83%–11.44%). In the case of intensity-based methods, we found that the larger the intensity value, the lower the angular error. Specifically, the performance of $I_{high}$ (i.e., an error of 10.11) was comparable with that of $G_{low}$ and $G_{high}$ (i.e., errors of 10.12, and 10.11, respectively). On the other hand, the performance of the intensity adjustment method was also lower as the illumination became darker, similar to the other methods. These results can be interpreted to mean that the intensity adjustment methods failed to enhance the appearance of images of eyes, such that the overall gaze estimation performance was degraded compared with the other enhancement methods. Finally, we could confirm our hypothesis H4 that the baseline methods cannot be applied to practical use-cases, such as a digital gallery or an art exhibition hall, where various lighting conditions are necessary. This is because the baseline methods adjust the illumination in a fixed way (e.g., by increasing the intensity by a particular value), compared to the proposed method which adaptively enhances the illumination of image.

**Figure 13:**
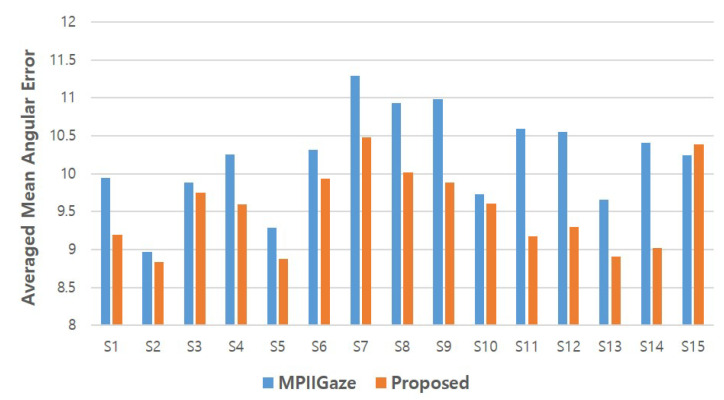
Mean gaze angular error for each subject of the MPIIGaze protocol and the proposed method.

Next, we provide subject-level details of the experimental results for each method. The error score reported here is the mean angular error averaged across all test sets. Figure 13 presents the subject-level results of the MPIIGaze protocol and the proposed method. Overall, the proposed method outperformed the MPIIGaze protocol for most of the subjects. However, significant differences between the performance of the MPIIGaze protocol and the proposed method did not exist in the case of subjects S2, S3, and S10 (i.e., the errors differ by approximately 0.12 degrees). For the remaining subjects, the proposed method outperformed the MPIIGaze protocol with a moderate difference of 0.6–1.3 degrees. In the case of subject S15, the MPIIGaze protocol outperformed the proposed method with a slight improvement (i.e., 10.23 for MPIIGaze vs. 10.38 for the proposed method).

**Figure 14:**
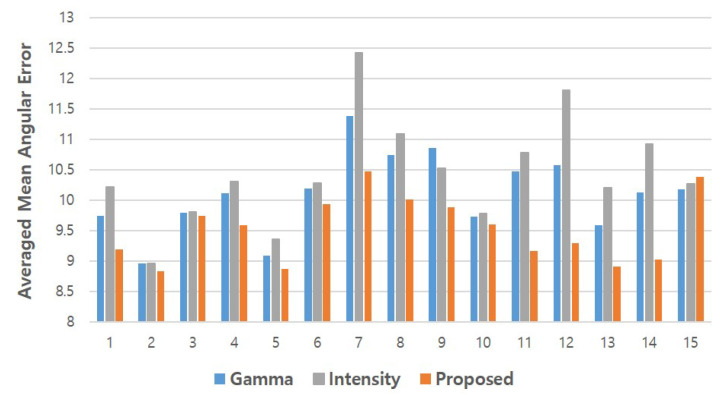
Mean gaze angular error for each subject of the proposed and baseline methods.

Figure 14 presents the averaged mean angular errors of the gamma correction methods (referred to as Gamma, the average of errors from $G_{low}$, $G_{mid}$, and $G_{high}$), the intensity adjustment methods (referred to as Intensity, the average errors from $I_{low}$, $I_{mid}$, and $I_{high}$), and the proposed method. The results closely resembled the findings presented in the previous Figure 13. First, similar to the previous result, the proposed method outperformed the baseline methods for most of the subjects. Second, significant performance differences did not exist between the baseline methods and the proposed method in the case of subjects S2, S3, and S10 (the difference between the proposed method and Gamma is 0.01–0.05, and between Intensity it is 0.06–0.18). For the remaining subjects, the proposed method outperformed both Gamma and Intensity with a moderate difference of 0.77 and 1.23 degrees on average, respectively. The baseline methods also outperformed the proposed method for subject S15. The angular error of the baseline methods was lower than that of the proposed method with differences of 0.12 (Intensity) and 0.21 (Gamma).

**Figure 15:**
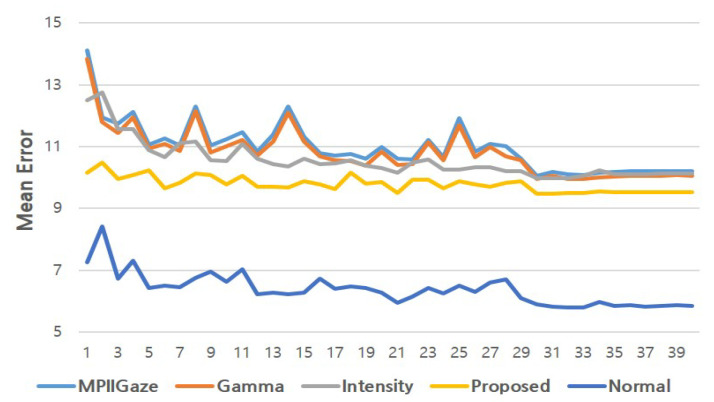
Changes in mean angular error according to the number of epochs.

Finally, we report the changes in the mean angular errors of each method according to training epochs in Figure 15. In this figure, Normal denotes the performance of the original MPIIGaze protocol on the original MPIIGaze test set. The errors produced by all the methods gradually decreased during the first 30 epochs and then converged. At the beginning of training, the MPIIGaze protocol and the baseline methods (i.e., Gamma and Intensity) produced high errors of 14.12, 13.82, and 12.49, respectively, whereas the error of the proposed method was relatively low (10.15). In the early stage of training, the average error of Intensity was lower than that of Gamma. However, at the end of training, Gamma outperformed both the MPIIGaze protocol and Intensity, as already summarized in Table 3, which confirms our hypothesis H5. Another noteworthy result is that the error curve of Normal and that of the proposed method behaved similarly. This can be interpreted to signify that the appearance of an image enhanced by the EnlightenGAN framework closely resembles that of the original normal-light images from the MPIIGaze data set.

## 5. Conclusions

In this study, we proposed an approach based on a generative adversarial network to improve the gaze estimation performance under low and dark light conditions. Our findings showed that the appropriate use of various methods to enhance images of eyes was able to improve the gaze estimation performance under low-light conditions. In particular, we validated that the performance of the proposed method based on EnlightenGAN is superior to that of the baseline methods with gamma correction or intensity adjustment. We conducted various experiments to demonstrate that the EnlightenGAN-based approach was able to adaptively enhance the appearance of images of the eye captured under low-light conditions and effectively recover the information of gaze features. By contrast, the baseline methods tried to modify the brightness of an image of the eye using a fixed intensity or gamma value. This caused the image to become hazy or noisy, which caused the performance to degrade.

However, the proposed approach still has limitations that would need to be addressed. First, the proposed method failed to perform effectively in certain cases (e.g., the low performance on subject S15), which means that training networks, data preprocessing, and data collection would have to be refined. Second, even though the average angular error of the proposed method is lower than that of the MPIIGaze protocol under low-light conditions, the performance of the proposed system is still insufficient to allow it to be successfully utilized in real-life environments and scenarios. Third, as all the reported results are from the evaluations with the test sets containing artificially darkened images only, the performance of the proposed approach in a more natural scenario, such as interactive environments with low illumination, is still unknown.

In future, we will extend our work in the following directions. First, we plan to study more advanced deep learning approaches to improve the gaze estimation performance under low-light conditions. Second, We also aim to extend our approach to other domains such as a more natural environment that would require a multiview setup or circumstances in which long-distance gaze estimation would be required. In this extended approach, we will introduce a multimodal approach (i.e., RGB, depth, and infrared image) for enhanced multi-user gaze estimation. Finally, since the previous datasets on gaze estimation mainly focus on daytime images, they are not suitable for gaze estimation research targeting interactive environments. To address this limitation, we are now studying how to collect images captured under more naturally low/dark-light conditions with various reflections. Moreover, the constructed dataset will be publicly available as a novel benchmark dataset that researchers can utilize to evaluate the performance of their own gaze estimators towards challenging conditions.

## Figures and Tables

**Table 1:** Mean error of the MPIIGaze protocol under various lighting conditions.

	Normal	${\mathrm{Low}}_{i}$	${\mathrm{Low}}_{g}$	Dark	${\mathrm{Low}}_{\mathrm{left}}$	${\mathrm{Low}}_{\mathrm{right}}$	${\mathrm{Dark}}_{\mathrm{left}}$	${\mathrm{Dark}}_{\mathrm{right}}$
Mean Error	5.76	9.9	9.71	10.56	10.4	10.2	10.4	10.22
Diff to Normal	-	+4.14	+3.95	+4.8	+4.64	+4.44	+4.64	+4.46

**Table 2:** Comparison of the performance between the MPIIGaze protocol and the proposed approach.

	${\mathrm{Low}}_{i}$	${\mathrm{Low}}_{g}$	Dark	${\mathrm{Low}}_{\mathrm{left}}$	${\mathrm{Low}}_{\mathrm{right}}$	${\mathrm{Dark}}_{\mathrm{left}}$	${\mathrm{Dark}}_{\mathrm{right}}$
Proposed	9.42	9.27	9.61	9.69	9.47	9.67	9.53
MPIIGaze	9.9	9.71	10.56	10.4	10.2	10.4	10.22
Improvement (deg)	0.48	0.44	0.95	0.71	0.73	0.73	0.69
Improvement (%)	4.8	4.53	8.9	6.8	7.15	7.01	6.75

**Table 3:** Comparison of mean errors between the proposed method and baseline methods.

	MPIIGaze	$G_{\mathrm{low}}$	$G_{\mathrm{mid}}$	$G_{\mathrm{high}}$	$I_{\mathrm{low}}$	$I_{\mathrm{mid}}$	$I_{\mathrm{high}}$	Proposed
$Low_{i}$	9.9	9.84	9.78	9.88	10.35	10.15	9.82	**9.42**
$Low_{g}$	9.71	9.62	9.63	9.56	10.04	9.94	9.71	**9.27**
Dark	10.56	10.48	10.39	10.47	11.66	11.14	10.54	**9.61**
$Low_{left}$	10.4	10.22	10.21	10.25	10.76	10.51	10.23	**9.69**
$Low_{right}$	10.2	10.13	10.10	10.14	10.72	10.51	10.09	**9.47**
$Dark_{left}$	10.4	10.21	10.15	10.28	10.87	10.59	10.21	**9.67**
$Dark_{right}$	10.22	10.36	10.16	10.25	10.86	10.46	10.19	**9.53**
Avg.	10.19	10.12	10.06	10.11	10.75	10.47	10.11	**9.52**
